# Autoantibodies frequency in children with visceral leishmaniosis

**DOI:** 10.1186/1546-0096-9-S1-P229

**Published:** 2011-09-14

**Authors:** Luciana Marques, Raquel Lisboa, Romana Maia

**Affiliations:** 1 Fortaleza University, Fortaleza, Brazil; 2 Faculdade de Medicina Christus, Fortaleza, Brazil

## Background

The visceral leishmaniosis (VL), or Calazar, is a chronic severe systemic disease, potentially fatal to humans. Currently, VL is the prototype of a specific immune dysfunction resulting from parasitism of leishmania donovani in macrophages, producing a broad spectrum of clinical and immunological reversible only with specific treatment. Serum Analysis from infected adult patients demonstrated the presence of autoantibodies against cellular and humoral components, and circulating immune complexes.

## Aim

To identify the profile of autoantibodies in pediatric patients with VL and its correlation with clinical outcome.

## Methods

Through a transversal study, was investigated the occurence of autoantibodies ( antinuclear antibodies (ANA), anti-DNA , anti-SM , anti-RNP , anti-SSb , anti-SSa. lupus anticoagulant, IgG and IgM anticardiolipin (aCL) antibodies ) in 14 patients (under 18 years) with diagnosis of VL, in the period October 2010 to March 2011.

## Results

The incidence of autoantibodies present in patients with VL was 42,8% 1 with ANA positive (7,1%), 2 with lupus anticoagulant antibodies positive (14,2%), 3 with IgM aCL antibodies positive (21,42%) and 2 with IgG aCL antibodie positive (14,2%). It were also found associated infections: sepsis, pneumonia and urinary tract infection in 71,42% of total patients. All patients with autoantibodies positive had also infections associated.

## Conclusion

Visceral leishmaniasis appears to association positively with the presence of ANA, lupus anticoagulant, IgG ang IgM aCL, in children, as in adults possibly by triggering a systemic humoral response of Th2. The association with other infections was present in all patients with positive autoantibodies.

